# Suicidal Ideations and Behavior in Patients With Young and Late Onset Dementia

**DOI:** 10.3389/fneur.2021.647396

**Published:** 2021-07-27

**Authors:** Marion Ortner, Lina Riedl, Ralf J. Jox, Julia Hartmann, Carola Roßmeier, Bianca Dorn, Victoria Kehl, Silvia Egert-Schwender, Julia Fischer, Janine Diehl-Schmid

**Affiliations:** ^1^Department of Psychiatry and Psychotherapy, School of Medicine, Klinikum rechts der Isar, Technical University of Munich, Munich, Germany; ^2^Institute of Humanities in Medicine, Lausanne University Hospital and University of Lausanne, Lausanne, Switzerland; ^3^Chair in Geriatric Palliative Care, Department of Medicine, Lausanne University Hospital and University of Lausanne, Lausanne, Switzerland; ^4^School of Medicine, Klinikum rechts der Isar, Institute for Medical Informatics, Statistics and Epidemiology, Technical University of Munich, Munich, Germany; ^5^Münchner Studienzentrum, Technical University of Munich, Munich, Germany; ^6^Munich Cluster for Systems Neurology, Munich, Germany

**Keywords:** suicidal ideations, suicide attempts, dementia, Alzheimer's disease, C-SSRS, late onset dementia, young onset dementia

## Abstract

**Background and Objectives:** Data on suicidal ideation, behavior and the risk factors in patients with dementia is scarce. To evaluate the prevalence of death wishes, suicidal ideation, and suicidal behavior of young (YOD) and late onset dementia (LOD) and to identify risk factors for suicidal ideation and behavior.

**Methods:** We interviewed 157 family caregivers of patients with advanced dementia using questions from the Columbia-Suicide Severity Rating Scale to gather information about suicidal ideation and behavior before the onset of symptoms of dementia, after the onset of dementia and within 30 days prior to the interview. At the time of the interview, we also assessed disease severity, cognitive function, and other psychological, behavioral and physical symptoms of the patients as well as the caregivers' psychological well-being.

**Results:** Forty four (28%) of the patients expressed suicidal ideation or behavior at some time after the onset of symptoms, and 14 (9%) of these within the month prior to the assessment. Two patients had attempted suicide after the onset of dementia. There were no statistically significant differences between patients with and without suicidal ideations or behavior with regards to demographics or age at onset of dementia. In patients with advanced dementia, Alzheimer's disease (rather than frontotemporal lobar degeneration), better cognitive function, more severe psychological, behavioral, and physical symptoms, and a reduced quality of life were associated with the expression of suicidal ideation.

**Conclusions:** According to caregivers' reports, majority of patients with dementia did not express suicidal ideation or show suicidal behavior. Patients who expressed suicidal ideation during early stages of dementia often stopped expressing them in advanced stages. It remains unclear if this was due to reduced communication abilities, a reduction of disease awareness, and/ or an adjustment to their situation.

## Introduction

Knowledge about death wishes, suicidal ideation and suicide attempts in patients with dementia is scarce and information about their prevalence and severity varies vastly. One challenge for researchers is the lack of uniform nomenclature (1, 2). Whereas, the National Institute of Mental Health defines suicidal ideation as “thinking about, considering, or planning suicide” (3), some authors further differentiate between active suicidal ideation involving thoughts and plans of killing oneself and passive suicidal ideation involving thoughts of or wishing for one's death without thoughts of killing oneself (4). Scales to assess the severity of suicidal ideation often encompass both passive and active suicidal ideation (5, 6).

While 5–10% of patients with very mild to severe dementia are reported to feel their “life not worth living” on the Hamilton Depression Rating Scale (7, 8), the number of patients with explicit death wishes or thoughts of suicide was markedly smaller. Draper et al. (7) described death wishes in 3% of patients and suicidal ideations or gestures in 0.9%. When interviewing patients and caregivers, Harwood and Sultzer (8) did not see patients with “wishes to be dead” or having actual “suicidal ideations or gestures.”

In a study by Koyama et al. (9), 10% (*n* = 64) of caregivers of outpatients with dementia answered with “yes” to the sub-question “does the patient express a wish for death or talk about killing himself/herself?” of the Neuropsychiatric Inventory (NPI) depression/dysphoria item (10). Interestingly, when assessing both self-reported and observer-reported suicidal ideation in the same group of patients, numbers diverge: While 2% of patients with mild Alzheimer's dementia self-reported suicidal thoughts, 15% of their caregivers reported that the patients had thought about suicide (11).

The actual risk of suicide attempts in dementia is considered to be low and not to exceed that of the age-matched general population (12). A Swedish study by Wiktorsson et al. (13) did not find a statistically significant association between suicide attempts among patients aged 70 or older and dementia. Annor et al. (14) investigated suicide in patients with dementia in Georgia, USA. They identified 91 patients with dementia that died by suicide between 2013 and 2016. Compared to the annual suicide rate in the US for persons 65 years and older of 16.8 per 100,000 population, the overall suicide rate among persons with dementia was 9.3 per 100,000 person-years. However, during the first 12 months after diagnosis, this number was almost 50 times as high (424.5/100,000 person-years). Then again, there are other studies reporting larger proportions of attempted and completed suicide among patients with dementia. In an Israeli study 7.4% and in a Chinese study 12% of admissions of demented patients to a gerontopsychiatric unit were due to suicide attempts (15, 16). In a sample consisting solely of US veterans, Seyfried et al. (17) observed that 0.09% (*n* = 241) of almost 300,000 veterans diagnosed with dementia died by suicide over a period of 5 years.

Significant psychiatric comorbidities, such as depression (7, 12, 17, 18), anxiety (8, 17), delusions (16), and alcohol or drug misuse (19), are considered risk factors for suicide attempts in patients with dementia (20). Other putative risk factors are recent diagnosis of dementia (14, 17), male sex (14), white race (17) and a symptom onset or diagnosis before the age of 65 years (14, 21). A large study among US veterans found a significantly higher prevalence of suicide ideations (4%) and plans or attempts (0.5%) in patients with frontotemporal lobar degeneration (FTLD) compared to other types of dementia (22). While a number of studies identified depression as a risk factor (7, 12, 17, 18), in their study of suicides among persons with dementia in Georgia, Annor et al. (14) found that depression was not a significant predictor of suicide, even though depressed mood was present in 38.7% of suicide completers prior to suicide. In contrast to psychiatric comorbidity, the severity of medical comorbidity does not seem to increase suicide risk (14, 17).

Considering the heterogeneity regarding the investigated cohorts (e.g., whole population, veterans only, psychiatric inpatients only, age, causes for dementia, severity of dementia, observed period of time) and the definition of “suicidal ideation,” which ranged from thinking one's life is no longer worth living to expressing the wish to kill oneself (7–9), it comes as no surprise that some of the mentioned epidemiological studies yielded inconsistent results. Symptoms were assessed by a variety of raters, ranging from patients themselves over family caregivers and nursing staff to psychiatrists. In consequence, different instruments were used to assess suicidal ideation and behavior and raters had different levels of experience in assessing and distinguishing between passive death wishes, active suicidal ideation and behavior, and actual suicide risk. In addition, the periods of time for which suicidal ideation or behavior was assessed differed between studies (7, 8). A particular problem when investigating suicidal ideation and behavior is that patients themselves might be reluctant to reveal symptoms. This might be due to shame, protection of family members, or fear of consequences, e.g., the admission to a psychiatric hospital. An additional challenge is that persons with dementia might not remember what they thought or intended to do between visits (23). In order to avoid this reporting bias, we decided to ask family caregivers of patients with advanced dementia about the patients' suicidal ideations and behavior. We did this retrospectively not only for suicidal ideation or behavior throughout the course of the disease, but also for the time before the onset of first symptoms of dementia. These caregiver reports were gathered within the prospective cohort study EPYLOGE [IssuEs in Palliative care for people in advanced and terminal stages of Young-onset (<65 years) and Late-Onset dementia in Germany] that assessed palliative care issues in patients with advanced dementia by patient exams, caregiver interviews and file research (24). Using data from EPYLOGE had the advantage that our sample consisted of almost equal numbers of subjects with young–onset dementia (YOD), defined as dementia with an onset of symptoms before the age of 65, and late-onset dementia (LOD).

The aims of our study were (1) to evaluate the prevalence of death wishes, suicidal ideation, and suicidal behavior throughout the course of young and late onset dementia, using retrospective family caregiver interviews; (2) to investigate the differences between patients with and without death wishes or suicidal ideation in terms of demographic characteristics, cognitive impairment, behavioral, psychological and physical symptoms and caregiver variables on the presence of suicidal ideation and behavior in patients with advanced dementia.

## Materials and Methods

### Ethics Statement

All assessments took place within the EPYLOGE-study (24). The study protocol was approved by the ethics committee of the Faculty of Medicine of the Technical University of Munich, Munich, Germany (reference number 281/17) and has been registered in ClinicalTrials.gov (NCT03364179). Patients and/or their legal surrogates and the family caregivers were required to provide written informed consent prior to any study specific procedures. All clinical investigations were conducted in accordance with the principles of the Declaration of Helsinki, seventh revision.

### Recruitment and Study Design

The data presented here was obtained from dyads of patients with advanced dementia, defined by a CDR global score of 2 or 3, and their family caregivers during the EPYLOGE study between December 2017 and April 2019. The EPYLOGE study protocol has been published previously (24). EPYLOGE participants were recruited from patients who had been diagnosed with neurodegenerative dementia at the Center of Cognitive Disorders at the University Hospital of the Technical University of Munich after 2005 and who suffered from advanced dementia at the time of EPYLOGE recruitment. At the EPYLOGE study assessment, demographic and medical information was collected, and several neuropsychological scales and questionnaires were administered. Among others, these included the Mini-Mental State Examination (MMSE) (25), the Clinical Dementia Rating Scale (CDR) global score (26) and Sum of Boxes (CDR-SOB) (27) as well as the FTLD-specific CDR (28), the Global Deterioration Scale for Assessment of Primary Degenerative Dementia (GDS) (29) for the patient and for the caregiver the Beck Depression Inventory II (BDI-II) (30), the 5-item World Health Organization well-being index (WHO-5) (31), and the Caregiver Strain Index (CSI) (32). Further information about the patient was obtained from the caregiver by administering the End-of-Life in Dementia Scale - Symptom Management (EOLD-SM) (33), the Quality of life in late-stage dementia (QUALID) (34), and the Neuropsychiatric Inventory (NPI) (10).

To assess suicidal ideation and behavior, the caregivers were interviewed with a modified version of the Columbia-Suicide Severity Rating Scale (C-SSRS) (6): instead of the patients, the caregivers were asked to answer the individual C-SSRS-questions on behalf of the patient either with “yes,” “no” or “do not know” for the time (i) prior to the onset of symptoms of dementia, (ii) after the onset of symptoms of dementia until 30 days prior to assessment, and (iii) within the last 30 days prior to the assessment. While in the original version of the C-SSRS the severity of suicidal ideation is assessed, we did not ask the caregivers to estimate the intensity of suicidal ideations in the patient at any given time. In detail, caregivers were first asked up to five questions from the C-SSRS to clarify whether suicidal ideation was present in the patient at any of the individual time points. Specifically, they were asked about (1) Wish to be dead; (2) Non-specific active suicidal thoughts, e.g., thoughts of killing himself/herself; (3) Active suicidal ideation with any methods but without a specific plan or intent; (4) Active suicidal ideation with some intent to act but without a specific plan; (5) Active suicidal ideation with a specific plan and intent. Questions about suicidal behavior covered preparatory acts and attempts (actual, interrupted, and aborted). If questions 1 & 2 were answered with “No,” questions 3–5 were skipped and the interview was continued with the suicidal behavior section.

### Statistical Analyses

The collected data was entered into a database especially created for the EPYLOGE study and independently monitored according to a study-specific manual and GCP-compliant monitoring standard operating procedures.

Group comparisons were performed as appropriate using the independent samples *t*-test for normally distributed continuous variables, Mann-Whitney U test for non-normally distributed variables, and χ2 test or Fisher's exact test for categorical variables. IBM SPSS statistics 25.0 software (IBM Corporation, Armonk, New York, United States) was used for all statistical analyses. All tests were performed two-sided at the explorative 5% significance level. We used the Benjamini-Hochberg procedure with an assumed false discovery rate (FDR) of 0.1 to adjust for multiple comparisons.

## Results

### Patients

**Figure 1:**
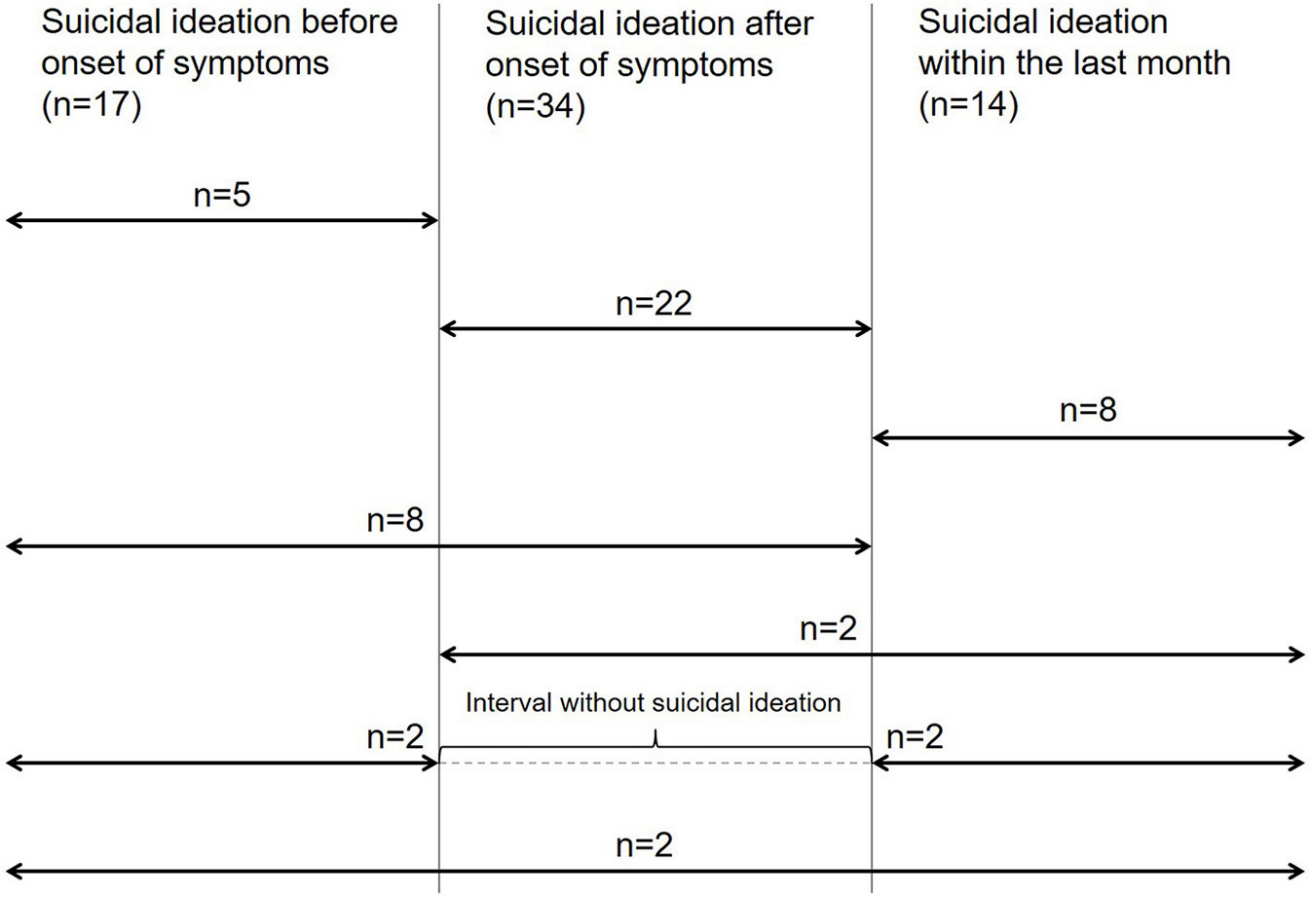
Suicidal ideations and behavior before onset of dementia and throughout the disease course. *n* = number of patients with suicidal ideations for the individual periods of time.

Demographics are described in Table 1. At time of the assessment, dementia was moderate (CDR global score = 2) in 3.2% (*n* = 5) of patients and severe (CDR global score = 3) in 96.8% (*n* = 152) of the patients. Information about suicidal ideation and attempts was gathered for 157 patients. According to their relatives, 49 (31.2%) of these patients expressed suicidal ideation or behavior as defined by the C-SSRS at any time. Before symptom onset, only 3.2% (*n* = 5) of subjects expressed suicidal ideation or behavior, while 14% (*n* = 22) expressed it after the onset of symptoms, but not within the last month prior to the interview, and 5.1% (*n* = 8) only during the month before the interview. 8.9% (*n* = 14) subjects expressed suicidal ideation at more than one period of time. The average time between the onset of symptoms and the assessment was 8.1 years, and 5.7 years between diagnosis and assessment. For distribution of suicidal ideations or behavior over the individual periods of time, see Figure 1.

**Table 1:** Patient demographic data.

**Variable**	**All subjects**
**Numbers (%)** **Mean ± SD (range) for normally distributed data** **Median (range) for non-normally distributed data**	***n* = 157**
Type of dementia AD:FTLD:VD:other	100:40 :4:13 (63.7:25.5:2.5:8.3%)
Sex (male:female)	70:87 (44.6:55.4%)
YOD:LOD	76:81 (51.6:48.4%)
Age at onset of symptoms (years)	65.18 ± 11.99 (27–96)
Age at time of diagnosis (years)	67.58 ± 11.67 (33–97)
Age at assessment (years)	73.32 ± 11.36 (40–101)
Formal education (years) (*n* = 149)	12 (5–25)
Living arrangements (home care:long term care)	70:87 (44.6:55.4%)
Religion (Christian:Muslim:none)	126:2:29 (80.3%:1.3%:18.5%)
Marital status (married:widowed:divorced:single)	104:32:15:8 (66.2:20.4:9.6:3.8%)
MMSE (*n* = 148)	0 (0–13)
CDR global score	3 (2–3)
CDR sum of boxes	18 (11–18)

*SD, standard deviation; n, number of subjects; AD, Alzheimer's disease; FTLD, frontotemporal lobar degeneration; VD, vascular dementia; MMSE, mini-mental state examination; CDR, clinical dementia rating scale; YOD, young onset dementia; LOD, late onset dementia*.

Two patients attempted suicide before and two patients after the onset of symptoms of dementia. There were no suicide attempts during the month preceding the assessment.

### Suicidal Ideation and Behavior After the Onset of Symptoms

Forty four out of 157 patients (28.0%) showed suicidal ideations or behavior at some time after the onset of symptoms (see Figure 1). Out of these, 30 patients (68.2%) had suicidal ideations after the onset of symptoms but not during the month prior to the interview (*n* = 13 for passive suicidal ideation; *n* = 15 for active suicidal ideation; *n* = 2 for suicide attempt). Patients with passive suicidal ideation expressed the wish to die and that life was no longer worth living (“I don't want to live any more…; I can't go on…; If I could just die…, this is no life…”). Patients with active suicidal ideation stated one or more potential methods they were contemplating in order to commit suicide. In detail, four considered hanging themselves, two wanted to jump off the balcony, two thought about shooting themselves, one patient considered suffocation, one getting hit by a car, one thought about lying down on the train tracks, one contemplated taking sleeping pills together with her husband, and one patient briefly considered physician assisted suicide in Switzerland. For two patients the caregivers said that they had expressed serious plans without stating specifics. Two out of the 30 patients attempted suicide as described in Section Suicide Attempts at Any Time Patients with active suicidal ideation stated one or more potential methods they were contemplating in order to commit suicide. In detail, four considered hanging themselves, two wanted to jump off the balcony, two thought about shooting themselves, one patient considered suffocation, one getting hit by a car, one thought about lying down on the train tracks, one contemplated taking sleeping pills together with her husband, and one patient briefly considered physician assisted suicide in Switzerland. For two patients the caregivers said that they had expressed serious plans without stating specifics. Two out of the 30 patients attempted suicide as described in section Suicide Attempts at Any Time.

### Suicidal Ideation and Behavior in the Month Prior to the Assessment

According to the caregivers, 14 out of 157 patients (8.9%) with advanced dementia had expressed suicidal ideations during the month preceding the study assessment (*n* = 10 for passive suicidal ideation; *n* = 4 for active suicidal ideation). Four of them had already had suicidal ideations previously in life before the onset of symptoms of dementia. None of the four patients who thought more actively about suicide had stated definite plans or actually attempted suicide [e.g., “if I knew how my sister killed herself…, please help me and give me something (to end my life)”]. Ten (6.4%) out of 157 caregivers felt unable to judge whether suicidal ideation was present during the month prior to the interview.

### Suicide Attempts at Any Time

Of the two patients attempting suicide before the onset of symptoms, one took sleeping pills with alcohol more than 25 years before the onset of symptoms and the other patient took pills (substance unknown) and went to the rooftop of a high-rise building and considered jumping. After the onset of dementia, one patient regularly drank copious amounts of alcohol with the intention of harming and ultimately killing herself and the other told her husband that she had eaten an unknown amount of belladonna berries she had collected in the forest. Her husband was unsure, however, if she had actually taken any belladonna berries as she had not experienced any symptoms. At the time of the assessment, both suicide attempts dated back ~10 years and took place about 2 years after the respective diagnosis of frontotemporal lobar degeneration (FTLD) or Alzheimer's disease (AD). Both patients were female and neither had voiced suicidal ideations previously in their lives.

### Comparisons Between Patients With and Without Suicidal Ideation or Behavior

There were no statistically significant differences between patients with and without suicidal ideation or behavior when looking at the entire period after the onset of dementia symptoms with regards to age, age at onset, age group (YOD vs. LOD), sex, and type of dementia (Supplementary Table 1).

When focusing on suicidal ideations within the month prior to the assessment, the distribution of the type of dementia differed between the two groups: those with suicidal ideation had a higher proportion of dementia due to Alzheimer's disease than those without suicidal ideation (*p* = 0.046) (Table 2). Patients with suicidal ideations had less years of formal education and were cognitively less impaired, e.g., the performed better on tests indicative for severity of dementia and cognitive impairment (CDR-SOB, CDR score for judgment and problem solving, FTLD-specific CDR language score, GDS, MMSE). At the same time they had more behavioral and psychological symptoms (higher scores on the NPI total score and NPI items for delusions, hallucinations, depression, anxiety, irritability, and appetite disturbance) and scored worse on scales measuring symptom management and quality of life (lower scores on the EOLD-SM and higher scores on the QUALID) (Tables 2, 3). There were no statistically significant differences regarding sex, YOD vs. LOD, age at onset of dementia, age at diagnosis, age at assessment, religion, or place of care (home care vs. long-term care) (Table 2). After applying the Benjamini-Hochberg procedure with a FDR of 0.1 the type of dementia, CDR-SOB, CDR score for judgment and problem solving, and the NPI items for anxiety and appetite disturbance were no longer statistically significant.

**Table 2:** Patients with and without suicidal ideations during the month before the assessment.

**Variable** ** mean ± SD (range)** ** median (range)** ** or n (%)**	**Suicidal ideations during the last month *n* = 14**	**No suicidal ideations during the last month** ** n = 133**	***p*-value**
Type of dementia (AD:FTLD:VD:other) (*n*)	11:0:1:2 (78.6:0:7.1:14.3%)	85:35:3:10 (63.9:26.3:2.3:7.5%)	0.046^d^
Sex (male:female) (*n*)	3:11 (21.4%:78.6%)	60:73 (45.1%:54.9%)	0.154^c^
YOD:LOD (*n*)	5:9	67:66	0.402^c^
Age at onset (years)	68.43 ±12.36 (50–96)	64.90 ± 12.13 (27–95)	0.304^a^
Age at diagnosis (years)	70.00 ± 12.07 (53–97)	67.34 ± 11.82 (33–95)	0.425^a^
Age at assessment (years)	76.00 ± 11.75 (59–98)	72.99 ± 11.60 (40–101)	0.355^a^
CDR-global	3 (3–3)	3 (2–3)	0.512^b^
CDR-SOB	17 (16–18)	18 (11–18)	0.036^b^
CDR judgement and problem solving	3 (2–3)	3 (0–3)	0.045^b^
FTLD-specific CDR language score	2 (1–3)	3 (1–3)	<0.001^b^^*^
GDS	6 (6–7)	7 (5–7)	0.001^b^^*^
MMSE	3 (0–10) *n* = 10	0 (0–13) *n* = 128	0.005^b^^*^
EOLD-SM	28 (13–35)	35 (5–57)	<0.001^b^^*^
QUALID	27 (17–44)	19 (9–37)	<0.001^b^^*^
Formal education (years)	10.5 (8–20)	12 (5–25) *n* = 126	0.005^b^^*^
Living arrangements (home:long term care) (*n*)	9:5 (64.3:35.7%)	58:75 (43.6:56.4%)	0.165^c^
Religion (Christian:Muslim:none) (*n*)	13:0:1 (92.9%:0%:7.1)	104:2:27 (78.2%:1.5%:20.3%)	0.432^d^
Marital status (married:widowed:divorced: single) (*n*)	9:4:1:0 (64.3:28.6:7.1:0.0%)	88:26:13:6 (66.2:19.5:9.8:4.5%)	0.901^d^

a *t-test for normally distributed and metric data*.

b *Mann-Whitney U test for not normally distributed data*.

c *x^2^ for categorical data*.

d *Fisher's exact test*.

* *Remained significant after Benjamini-Hochberg procedure with an assumed FDR of 0.1*.

*In 10 cases (6.4%) the caregivers felt unable to assess if suicidal ideation was present or not during the month prior to the assessment*.

*SD, standard deviation; n, number of subjects; AD, Alzheimer's disease; FTLD, frontotemporal lobar degeneration; VD, vascular dementia; YOD, young onset dementia; LOD, late onset dementia; CDR, clinical dementia rating scale; SOB, sum of boxes; GDS, global deterioration scale; MMSE, mini-mental state examination; EOLD-SM, end-of-life in dementia scale; QUALID, quality of life in late stage dementia*.

**Table 3:** NPI-scores of patients with and without suicidal ideations during the month before the assessment.

**Variable** ** mean ± SD (range)** ** median (range)** ** or *n***	**Suicidal ideation during the last month *n* = 14**	**No suicidal ideation during the last month** ***n* = 133**	***p*-value**
NPI total score	31 (14–79)	20 (0–76)	0.005^a^^*^
NPI—delusions	0 (0–12)	0 (0–12) *n* = 93	0.002^a^^*^
NPI—hallucinations	1.5 (0–12)	0 (0–8) *n* = 96	<0.001^a^^*^
NPI—aggressiveness	1 (0–9)	0 (0–12) *n* = 130	0.160^a^
NPI—depression	2 (0–12) *n* = 13	0 (0–12) *n* = 122	0.001^a^^*^
NPI—anxiety	1 (0–12)	0 (0–12) *n* = 131	0.037^a^
NPI—euphoria	0 (0–0)	0 (0–12) *n* = 131	0.286^a^
NPI—apathy	8 (0–12)	8 (0–12) *n* = 119	0.840^a^
NPI—disinhibition	0 (0–6)	0 (0–12) *n* = 122	0.996^a^
NPI irritability	1.5 (0–12)	0 (0–12) *n* = 125	0.016^a^^*^
NPI—motor disorders	4 (0–12)	4 (0–12) *n* = 126	0.849^a^
NPI—sleep disorder	0 (0–9)	0 (0–12) *n* = 132	0.930^a^
NPI—appetite disturbance	0 (0–12) *n* = 13	0 (0–12) *n* = 68	0.032^a^

a *Mann-Whitney U test for not normally distributed data*.

* *Remained significant after Benjamini-Hochberg procedure with an assumed FDR of 0.1*.

*In 10 cases (6.4%) the caregivers felt unable to assess if suicidal ideation was present or not during the month prior to the assessment*.

*SD, standard deviation; n, number of subjects; NPI, neuropsychological inventory*.

Caregivers who reported the patient had suicidal ideations during the month prior to the study assessment spent significantly more time (i.e., days per month) with the patients than those who did not report suicidal ideations (*p* = 0.019). After applying the Benjamini-Hochberg procedure this result was no longer statistically significant. There were no statistically significant differences regarding sex, age, education (Table 1), caregiver strain, or depression in caregivers of patients with suicidal ideation during the month prior to the assessment compared to caregivers of patients without suicidal ideation (Supplementary Table 2).

### Comparison Between Patients With Young Onset Dementia and Late Onset Dementia

Patients with YOD were significantly younger than patients with LOD (*p* < 0.001, Table 4). There was no difference regarding suicidal ideation during any period of time between YOD and LOD. The distribution of type of dementia differed significantly with more FTLD patients in the YOD cohort and more AD-patients in the LOD cohort (*p* = 0.006). In a logistic regression analysis with suicidal ideation as dependent and age at onset (YOD/LOD) and FTLD (yes/no) as independent variables, the variables and their interaction was not significant. At the time of the study assessment patients with YOD scored significantly worse in the FTLD-specific CDR language score (*p* = 0.007) and the NPI disinhibition item (*p* = 0.007). There were no significant differences regarding CDR global score, CDR-SOB, NPI total score, or other neuropsychological measures. For details, see Table 4.

**Table 4:** Patients with young and late onset dementia.

**Variable** ** mean ± SD (range)** ** median (range)** ** or *n* (%)**	**YOD**	**LOD**	***p*-value**
Number of patients (n)	76	81	
Type of dementia (AD:FTLD:VD:other)	45:27:0:4 (59.2:35.5:0.0:5.3%)	55:13:4:9 (67.9:16.0:4.9:11.1%)	0.006^d^^*^
Sex (male:female)	40:36 (52.6:47.4%)	30:51 (37.0:63.0%)	0.055^c^
Age at onset (years)	55.16 ± 7.32 (27–64)	74.59 ± 6.69 (65–96)	<0.001^a^^*^
Age at diagnosis (years)	57.99 ± 7.49 (33–71)	76.58 ± 6.59 (66–97)	<0.001^a^^*^
Age at assessment (years)	64.34 ± 8.11 (40–82)	81.75 ± 6.44 (70–101)	<0.001^a^^*^
Suicidal ideation before onset of symptoms yes:no	5:70 (6.7:93.3%) *n* = 75	4:75 (5.1:94.9%) *n* = 79	0.741^d^
Suicidal ideation any time after the onset of symptoms yes:no	17:59 (22.4:77.6%)	26:55 (32.1:67.9%)	0.211^c^
Suicidal ideation during the last month yes:no	5:67 (6.9:93.1%) *n* = 72	9:66 (12.0:88.0%) *n* = 75	0.402^c^
CDR-global	3 (2–3)	3 (2–3)	0.152^b^
CDR-SOB	18 (11–18)	18 (12–18)	0.144^b^
CDR judgement and problem solving	3 (2–3)	3 (0–3)	0.753^b^
FTLD-specific CDR language score	3 (1–3)	3 (1–3)	0.007^b*^
GDS	7 (5–7)	7 (6–7)	0.345^b^
MMSE	0 (0–13)	0 (0–13)	0.137^b^
NPI total score	21.5 (0–72)	20 (0–79)	0.712^b^
EOLD-SM	34.5 (10–45)	35 (5–57)	0.987^b^
QUALID	19 (9–40)	20 (12–44)	0.150^b^
Formal education (years)	12 (5–25)	11 (7–25)	0.154^b^
Living arrangements (home:long term care)	32:44 (42.1:57.9%)	38:43 (46.9:53.1%)	0.630^c^
Religion (Christian:Muslim:none)	55:2:19 (72.4:2.6:25.0%)	71:2:10 (87.7:0.0:12.3%)	0.027^d^^*^
Marital status (married:widowed: divorced:single)	61:3:9:3 (80.3:3.9:11.8:3.9%)	43:29:6:29 (53.1:35.8:7.4:3.7%)	<0.001^d^^*^

a *t-test for normally distributed and metric data*.

b *Mann-Whitney U test for not normally distributed data*.

c *x^2^ for categorical data*.

d *Fisher's exact test*.

* *Remained significant after Benjamini-Hochberg procedure with an assumed FDR of 0.1*.

*In 10 cases (6.4%) the caregivers felt unable to assess if suicidal ideation was present or not during the month prior to the assessment*.

*SD, standard deviation; YOD, young onset dementia; LOD, late onset dementia; n, number of subjects; AD, Alzheimer's disease; FTLD, frontotemporal lobar degeneration; VD, vascular dementia; CDR, clinical dementia rating scale; SOB, sum of boxes; GDS, global deterioration scale; MMSE, mini-mental state examination; EOLD-SM, end of life in dementia symptom management; QUALID, quality of life in late stage dementia*.

## Discussion

In our unique patient cohort of 157 patients with advanced dementia, 44 patients (28.0%) had—according to the family caregivers—expressed suicidal ideations at some time after the onset of dementia. Out of these 44 patients, 14 (8.9% out of 157 patients) had suicidal ideations within the month prior to the study assessment. Two patients (1.3%) had attempted suicide ~24 months after the diagnosis of dementia.

It might be considered surprising that 72% of the patients with dementia did not have suicidal ideations or behavior throughout the whole disease trajectory. This might only in part be caused by a reduced disease awareness at the time of diagnosis. An alternative explanation could be that—once in the situation—the perspective on the burden of the disease differs significantly from the perspective of those who are healthy. The majority of patients with dementia obviously do not think that their life with dementia is not worth living. Compared to our study, where 28% of patients were reported to have suicidal ideation at some time after diagnosis, up to 23.5% of patients with probable Huntington's disease and up to 39% of patients with amyotrophic lateral sclerosis were reported to have passive death wishes or active suicidal ideation (35, 36). In Parkinson's disease suicidal ideation has been reported in 22.7–30.2% (37, 38). When considering only active suicidal ideation, this number decreased to 11.2%, with 4.3% actually having attempted suicide (38). These numbers are similar to what was reported for our participants with overall suicidal ideation of 28.0%, active suicidal ideation being reported for 12.1%, and 1.3% having attempted suicide after the onset of symptoms. Compared to the general German population with an incidence of suicidal ideation of 8% (39), however, suicidal ideation is seen far more often in our patients and patients with Parkinson's disease. A nationwide, retrospective cohort study in Denmark investigated suicide in patients with neurological disorders between 1980 and 2016, and reported a fully adjusted incidence rate ratio of 4.9 for both amyotrophic lateral sclerosis and for Huntington's disease, of 1.7 for Parkinson's disease, and 0.8 for dementia (40).

Furthermore, it is remarkable that in advanced stages of the disease, when patients are actually more impaired and dependent, they less often express the wish to die. In our sample of patients with advanced dementia, suicidal ideation in the month prior to the study assessment was significantly more often reported for patients with a higher MMSE score and less impaired language skills. These patients might still have been aware of the disease and its consequences. At the same time they were still capable of expressing the wish to die. It remains unclear if the patients without suicidal ideations, who were cognitively more impaired, lacked the insight into their situation and/ or were no longer able to communicate their wish to die or were simply content with their situation. The latter hypothesis would be in line with the finding that patients with advanced dementia who expressed suicidal ideations suffered more often and more severely from delusions, hallucinations, depressive symptoms, anxiety, irritability and appetite disturbance measured with the NPI. Additionally, they scored worse on EOLD-SM and QUALID. Taken together, they suffered from more physical and psychological symptoms and had a lower quality of life than patients without suicidal ideations. It is not surprising, that patients who experience burdensome physical, behavioral and psychological symptoms value their lives as less worth living than patients with a high quality of life.

In this study, we used the term “suicidal ideation” as it is used in the C-SSRS, covering the spectrum from “*wishing to be dead or to fall asleep and never wake up again”* to “*having a concrete plan with the full intention to carry it out*”. In particular, the feeling that life is no longer worth living could also be interpreted as a sign of hopelessness and/ or depression rather than the actual wish to be dead or to end one's life. Even the initial wish to be dead might primarily be founded in a fear of the future and the wish to spare oneself a life with increasing limitations and one's family the emotional and financial burden that is likely to come, rather than the desire to actually be dead or end one's life.

When looking at the individual questions of the C-SSRS, 8.9% of the patients felt their life is not worth living, which is in accordance with literature, where this finding is reported for 5–10% of patients (7, 8). When focusing on the month prior to the assessment, this number decreased to 6.4%. More specific death wishes were reported for 9.6% of patients in our study at any time after diagnosis and 2.5% during the month prior to the assessment. Other studies found specific death wishes in 0% (8) to 3% (7) of patients with dementia. In these studies, however, it was not reported how long the patients had already suffered from dementia at the time of the assessment and if suicidal ideation had changed over time.

Some studies describe that up to 12% of admissions of patients with dementia to gerontopsychiatric units are due to suicide attempts (15, 16). However, it is important to keep in mind that this represents only the percentage of patients that needed to be admitted to a psychiatric hospital and not the percentage of suicide attempts in the entire cohort of patients with dementia, which is markedly lower. Caution is also advised when transferring the results from studies using data from US veterans, where 0.09% of patients diagnosed with dementia committed suicide, 73% of them by firearms (17). While some risk factors such as depression or inpatient psychiatric hospitalization are likely valid for most populations, the access to and knowledge about the use of firearms as a suicide method might be less prevalent in most other western countries with more restrictive gun regulations. Knowing how to properly handle and kill with firearms and having access to them might lower the threshold to use them to commit suicide.

An important finding of our study, which included a high proportion (48%) of patients with YOD, is that suicidal ideation did not differ between YOD and LOD at any time point after the onset of symptoms of dementia. This is surprising, since some studies have pointed out that YOD patients have higher levels of disease awareness, defined as the ability to acknowledge changes caused by deficits related to the disease process (41, 42).

While Lai et al. described a higher prevalence of suicidal ideations and attempts in patients with FTLD (22), in our study the prevalence of suicidal ideation was higher in patients with AD. The distribution of the types of dementia differed significantly between YOD and LOD, with a higher proportion of FTLD in the YOD group.

Where assisted suicide or euthanasia is a legal option, new challenges arise, especially in cases of advanced directives for euthanasia. Free will is mandatory in all countries allowing assisted dying and euthanasia.

The continuous decline of cognitive functions, including insight and language capacities, poses a challenge when assessing the patients' well-being, especially when it comes to death wishes and suicidal ideations. Impairments intensify with increasing severity of dementia until patients with advanced dementia are no longer capable of communicating their thoughts about death wishes or suicidal ideation clearly (21, 23). At the same time, as shown in our study, the prevalence of suicidal ideation appears to decrease in the advanced stages of dementia, possibly reflecting patients' “change of mind.”

This problem is very well-demonstrated by the case of a 74-year-old severely demented woman who had given advanced directive to receive euthanasia in case she could no longer live at home and deemed the time right to go (43–45). After she was admitted to a nursing home, she was euthanized with the consent and in the presence of her family in 2016. However, before the patient was euthanized she made inconsistent statements as to whether it was time for her to go or not. It was impossible for the doctor to re-evaluate if the patient still wished to be euthanized since the patient was no longer capable of grasping the concepts of suicide and euthanasia. The regional euthanasia review committee deemed the euthanasia to be not in accordance with the statutory due care criteria. After a criminal investigation, in September 2019 the doctor was acquitted of all charges as the court concluded that the doctor had administered euthanasia at the explicit and serious request of the patient (46).

## Limitations

A limitation of our study is that the study design did not allow for investigations of the prevalence of actual suicides. Further, the retrospective collection of information about suicidal ideation and behavior from the family caregivers might be considered as a limitation. One might argue that a lack of first-hand information from the patient weakens the relevance of the study. We are convinced, however, that caregiver interviews provide more reliable data at this late stage of dementia. Even so, it is possible that some caregivers reported no suicidal ideation when they were unable to judge if suicidal ideation was present or not. Patients are sometimes reluctant to share thoughts of death wishes or suicidal ideations with their physician out of fear of being admitted to a psychiatric institution. Additionally, as the patients suffered from advanced dementia at the time of the assessment, patients might not have been able to give reliable information about the whole time period since before disease onset. In a study with patients with mild Alzheimer's dementia by Rubin and Kinscherf (11), caregivers reported that the patient had suicidal thoughts seven times more often than the patients stated themselves (15 vs. 2%). It is unclear, however, if this discrepancy is caused by the patients' forgetfulness or if caregivers might project their view of the patients' situation and therefore over-report suicidal thoughts. This goes along with our finding that caregivers who spent more time per week with the patients in the month prior to the study assessment reported more suicidal ideations in the patients they cared for than the caregivers of patients whom they saw less frequently. A bias cannot be excluded to the effect that caregivers who saw the patients less often just had less opportunity to hear the patient express suicidal ideations.

## Conclusion

According to caregivers' reports, the majority of patients with dementia did not express suicidal ideations or show suicidal behavior throughout the disease course. Patients who initially expressed suicidal ideations often stopped expressing them in advanced disease stages. It remains unclear if this is because the patients are no longer capable of communicating their death wishes and/or because a reduction of disease awareness and/ or an adjustment with the situation causes the death wish to diminish or fade. Twenty eight percent of the patients in our study had suicidal ideations or showed suicidal behavior after the onset of dementia. It was beyond the scope of this work to evaluate if/ how these patients were counseled and treated. In any case, appropriate measures to reduce depressive symptoms, suicidal ideations and behavior should not be neglected—even or especially in patients with dementia.

## Data Availability Statement

The datasets used and/or analyzed during the current study are available form the corresponding author on reasonable request. However, due to the nature of psyeudonymised patient data, a material transfer agreement is required to meet ethical standards and data privacy laws of Germany.

## Ethics Statement

The studies involving human participants were reviewed and approved by Ethics committee of the Faculty of Medicine of the Technical University of Munich, Munich, Germany (reference number 281/17). It has also been registered in ClinicalTrials.gov (NCT03364179). The participants/patients (and/or their legal surrogates) provided their written informed consent to participate in this study.

## Author Contributions

MO, LR, JH, CR, and BD: acquisition and analysis of data and drafting a significant portion of the manuscript. RJ: formulating research questions, conception and design of the study, and revising the manuscript for intellectual content. VK: analysis of data and drafting a significant portion of the manuscript. SE-S: conception and design of the study, and revising the manuscript for intellectual content. JF: acquisition and analysis of data and revising the manuscript for intellectual content. JD-S: formulation of research questions, conception and design of the study, acquisition and analysis of data, and drafting a significant portion of the manuscript. All authors read and approved the final manuscript.

## Conflict of Interest

The authors declare that the research was conducted in the absence of any commercial or financial relationships that could be construed as a potential conflict of interest.

## Publisher's Note

All claims expressed in this article are solely those of the authors and do not necessarily represent those of their affiliated organizations, or those of the publisher, the editors and the reviewers. Any product that may be evaluated in this article, or claim that may be made by its manufacturer, is not guaranteed or endorsed by the publisher.
