# Supplementary material for: Suicidal Ideations and Behavior in Patients With Young and Late Onset Dementia
Source: Front Neurol. 2021 Jul 27;12:647396. doi: 10.3389/fneur.2021.647396 (PMC8353362; doi:10.3389/fneur.2021.647396)
Supplement: Supplementary file 1 [file Data_Sheet_1.docx]

**Supplementary Tables**

Supplementary Table 1: Patients with and without suicidal ideations since onset of dementia

Legend to Supplementary Table 1: SD: standard deviation; n: number of subjects; AD: Alzheimer’s disease; FTLD: frontotemporal lobar degeneration; VD: vascular dementia; YOD: young onset dementia; LOD: late onset dementia.
^a^: t-test for normally distributed and metric data; ^b^: Mann-Whitney U test for not normally distributed data; ^c^: x² for categorical data; ^d^: Fisher’s exact test.

| **Variable**  mean ± SD (range), median (range), or n (%) | Suicidal ideation since onset of symptoms (any time)  n=43 | **No** suicidal ideation since onset of symptoms (any time)  n=114 | p-value |
| --- | --- | --- | --- |
| Type of dementia  (AD : FTLD : VD : other) | 31 : 5 : 2 : 5 (72.1 : 11.6 : 4.7 : 11.6%) | 69 : 35 : 2: 8 (60.5 : 30.7 : 1.8 : 7%) | 0.066 ^d^ |
| Sex (male:female) | 17 : 26 (39.5% : 60.5%) | 53 : 61 (46.5% : 53.5%) | 0.475 ^c^ |
| YOD : LOD | 17 : 26 (39.5 : 60.5%) | 58 : 55 (51.8 : 48.2%) | 0.211 ^c^ |
| Age at onset [years] | 66.65 ±12.14 (38-96) | 64.63 ± 11.93 (27-95) | 0.353 ^a^ |
| Age at diagnosis [years] | 68.63 ± 12.51 (39-97) | 67.18 ± 11.37 (33-95) | 0.491 ^a^ |
| Age at assessment [years] | 74.33 ± 11.50 (41-98) | 72.95 ± 11.34 (40-101) | 0.500 ^a^ |
| Formal education [years] | 11 (5-20) | 12 (8-25) n=106 | 0.371 ^b^ |
| Living arrangements (home care : long term care) | 19 : 24 (44.2 : 55.8%) | 51 : 63 (44.7 : 55.3%) | 1.000 ^c^ |
| Religion (Christian : Muslim : none) | 35 : 1 : 7 ( 81.4% : 2.3% : 16.3%) | 91 : 1 : 22 (79.8% : 0.9% : 19.3%) | 0.659 ^d^ |
| Marital status (married : widowed : divorced : single) [n] | 28 : 10 : 3 : 2 (65.1 : 23.3 : 7.0 : 4.7%) | 76 : 22 : 12 : 4 (66.7 : 19.3 : 10.5 : 3.5%) | 0.810 ^d^ |

Supplementary Table 2

Legend to Supplementary Table 2: SD: standard deviation; n: number of subjects; WHO-5: 5-item World Health Organization well-being index; BDI-II: Beck depression inventory II; CSI: caregiver strain index.
^a^: t-test for normally distributed and metric data; ^b^: Mann-Whitney U test for not normally distributed data; ^c^: x² for categorical data; ^d^: Fisher’s exact test.
In 10 cases (6.4%) the caregivers felt unable to assess if suicidal ideation was present or not during the month prior to the assessment.

| **Variable**  mean ± SD (range),  median (range), or n (%) | **Caregiver reporting** suicidal ideation during the **last month**  n=14 | **Caregiver not reporting** suicidal ideation during the **last month**  n=133 | p-values |
| --- | --- | --- | --- |
| Sex caregiver (male/ female) [n] | 7 : 7 (50.0 : 50.0%) | 47 / 86 (35.3 : 64.7%) | 0.279 ^c^ |
| Age caregiver [years] | 64.50 ± 10.47 (48 - 78) | 64.81 ± 10.86 (33 - 88) | 0.918 ^a^ |
| Formal education caregiver [years] | 13 (11-20) | 13 (5-34) n=130 | 0.736 ^b^ |
| Religion caregiver (Christian : Muslim : none) [n] | 13 : 0 : 1 (92.9 : 0.0 : 7.1%) | 91 : 2 : 40 (68.4 : 1.5 : 30.1%) | 0.192 ^d^ |
| Marital status caregiver (married : widowed : divorced : single) [n] | 12 : 0 : 1 : 1 (85.7 : 0.0 : 7.1 : 7.1%) | 116 : 3 : 6 : 8 (87.2 : 2.3 : 4.5 : 6.0%) | 0.726 ^d^ |
| Relationship to the patient (spouse : parent : child : other) [n] | 8 : 0 :6 : 0 (57.1 : 0.0 : 42.9 : 0.0%) | 82 : 3 :36 : 12 (61.6 : 2.3 : 27.1 : 9.0%) | 0.652 ^d^ |
| Lives with patient (yes:no) [n] | 6 : 8 (42.9 : 57.1%) | 54:79 (40.6 : 59.4%) | 0.870 ^c^ |
| **Days spent with the patient during the last month [n]** | **18.5 (8-30) n=8** | **8 (1-30) n=78** | **0.019 ^b^** |
| Average time spent with the patient [hours/day/month] | 2  (0.50-4.00) n=8 | 2 (0.33-8.00) n=78 | 0.775^b^ |
| WHO-5 | 13 (2-20) n=13 | 14 (1-25) n=114 | 0.591 ^b^ |
| BDI-II | 14 (4-29) n=13 | 8 (0-28) n=112 | 0.298 ^b^ |
| CSI | 7.5 (3-12) | 6 (0-12) n=131 | 0.112 ^b^ |
